# Supplementary material for: Archived natural DNA samplers reveal four decades of biodiversity change across the tree of life
Source: Nat Ecol Evol. 2025 Aug 1;9(10):1873–84. doi: 10.1038/s41559-025-02812-6 (PMC12507656; doi:10.1038/s41559-025-02812-6)
Supplement: Supplementary file 1 — Reporting Summary [file 41559_2025_2812_MOESM1_ESM.pdf]

nature portfolio

Corresponding author(s): Henrik Krehenwinkel

Last updated by author(s): May 15, 2025

Reporting Summary

Nature Portfolio wishes to improve the reproducibility of the work that we publish. This form provides structure for consistency and transparency in reporting. For further information on Nature Portfolio policies, see our Editorial Policies and the Editorial Policy Checklist.

Statistics

For all statistical analyses, confirm that the following items are present in the figure legend, table legend, main text, or Methods section.

n/a	Confirmed	
☐	☒	The exact sample size (*n*) for each experimental group/condition, given as a discrete number and unit of measurement
☐	☒	A statement on whether measurements were taken from distinct samples or whether the same sample was measured repeatedly
☐	☒	The statistical test(s) used AND whether they are one- or two-sided<br>*Only common tests should be described solely by name; describe more complex techniques in the Methods section.*
☒	☐	A description of all covariates tested
☐	☒	A description of any assumptions or corrections, such as tests of normality and adjustment for multiple comparisons
☐	☒	A full description of the statistical parameters including central tendency (e.g. means) or other basic estimates (e.g. regression coefficient) AND variation (e.g. standard deviation) or associated estimates of uncertainty (e.g. confidence intervals)
☐	☒	For null hypothesis testing, the test statistic (e.g. *F*, *t*, *r*) with confidence intervals, effect sizes, degrees of freedom and *P* value noted<br>*Give P values as exact values whenever suitable.*
☒	☐	For Bayesian analysis, information on the choice of priors and Markov chain Monte Carlo settings
☒	☐	For hierarchical and complex designs, identification of the appropriate level for tests and full reporting of outcomes
☒	☐	Estimates of effect sizes (e.g. Cohen's *d*, Pearson's *r*), indicating how they were calculated

Our web collection on statistics for biologists contains articles on many of the points above.

Software and code

Policy information about availability of computer code

Data collection	All data analyzed in this study were generated for this purpose (see next section).
Data analysis	bioinformatic scripts. For data analyses and statistics, we used R 4.3.1. with the packages scales_1.3.0, phytools_2.3-0, maps_3.4.2, ape_5.8, betapart_1.6, RColorBrewer_1.1-3, nlme_3.1-164, ggplot2_3.5.1, vegan_2.6-6.1, lattice_0.22-5 and permute_0.9-7. We also used python 3 with the libraries numpy, keras and sklearn for training the artificial neural networks. All the scripts we used and the custom algorithms we developed (included the non-neutral model for community ecology and the simulations) are freely available to editors and reviewers following this private link https://www.scidb.cn/en/s/U7NFv2; DOI: 10.57760/sciencedb.13553 (see Code availability section).

For manuscripts utilizing custom algorithms or software that are central to the research but not yet described in published literature, software must be made available to editors and reviewers. We strongly encourage code deposition in a community repository (e.g. GitHub). See the Nature Portfolio guidelines for submitting code & software for further information.

Data

Policy information about availability of data

All manuscripts must include a data availability statement. This statement should provide the following information, where applicable:

- Accession codes, unique identifiers, or web links for publicly available datasets
- A description of any restrictions on data availability
- For clinical datasets or third party data, please ensure that the statement adheres to our policy

Raw Illumina sequencing data will be available in the ENA repository under the accession number PRJEB88877.
OTU tables, supplementary data, source data files for figures, and sample sizes underlying all box plots and means with error bars shown are available in the Science Data Bank (DOI: 10.57760/sciencedb.13553). Private link for editors and reviewers: https://www.scidb.cn/en/s/U7NFv2

Research involving human participants, their data, or biological material

Policy information about studies with human participants or human data. See also policy information about sex, gender (identity/presentation), and sexual orientation and race, ethnicity and racism.

Reporting on sex and gender	-
Reporting on race, ethnicity, or other socially relevant groupings	-
Population characteristics	-
Recruitment	-
Ethics oversight	-

Note that full information on the approval of the study protocol must also be provided in the manuscript.

Field-specific reporting

Please select the one below that is the best fit for your research. If you are not sure, read the appropriate sections before making your selection.

☐ Life sciences ☐ Behavioural & social sciences ☒ Ecological, evolutionary & environmental sciences

For a reference copy of the document with all sections, see nature.com/documents/nr-reporting-summary-flat.pdf

Ecological, evolutionary & environmental sciences study design

All studies must disclose on these points even when the disclosure is negative.

Study description	We used 38 years of archived samples of the German Environmental Specimen Bank, namely tree leaves (Fagus sylvatica, Picea abies and Populus nigra), mussel tissue (Dreissena polymorpha and Mytilus edulis) and coastal macroalgae (Fucus vesiculosus), as natural eDNA samplers to analyze retrospective biodiversity trends using a metabarcoding approach.<br>We looked at alpha as well as temporal, spatial beta and gamma-diversity patterns across the whole of Germany and compared them to the null-expectations of a non-neutral model for community ecology.<br>We used two to three primer pairs for each sampling species and performed three to six PCR replicates per sample to analyze metazoan, algal/protozoan, fungal and bacterial communties.
Research sample	We analyzed tree leaves (Fagus sylvatica, Picea abies and Populus nigra), mussel tissue (Dreissena polymorpha and Mytilus edulis) and coastal macroalgae (Fucus vesiculosus) since these species are routinely sampled by the German Environmental Specimen Bank (GESB) according to highly standardized sampling guidelines, which are publicly available on the homepage of the GESB. Samples have been perfectly preserved over liquid nitrogen since the time of collection.
Sampling strategy	Samples have been collected by the German Environmental Specimen Bank according to highly standardizes guidelines. No sample-size calculation was performed. All available samples were used for the analysis to achieve a time series as long and as complete as possible.
Data collection	Metabarcoding data was generated from Illumina sequencing at Trier University.
Timing and spatial scale	Earliest samples were collected in 1985 and latest in 2022. Sampling was performed annually or biannually.
Data exclusions	Some samples were excluded from the statistical analysis to achieve consistent time series. For details see Methods section "Statistical model and analyses of community diversity".

Reproducibility	The German Environmental Specimen Bank (GESB) uses highly standardized and publicly available sampling guidelines to secure sampling reproducibility. These guidelines can be found on the GESB homepage. For laboratory work we used established protocols for DNA isolation, DNA amplification and Illumina sequencing. For details see Methods section "DNA extraction, library preparation, sequencing and sequence processing" and supplementary Table 5 "Details of the laboratory workflow".
Randomization	Samples were allocated into groups according to sampling species, since they represent different ecosystems (marine, limnic and terrestrial). Tree species were allocated into one group, since they represent the terrestrial ecosystem. Additionally, trends for the terrestrial ecosystem are shown in Extended Data Figure 3 to provide detailed information for each tree species.
Blinding	Blinding was not relevant for this study since all data sets were treated equally.

Did the study involve field work? ☒ Yes ☐ No

Field work, collection and transport

Field conditions	Required field conditions depend on sampling species. Detailed information for each sampling species is listed in the respective GESB guideline.
Location	Species Location Latitude_WGS84 Longitude_WGS84<br>European Beech (Fagus sylvatica) Bayerischer Wald 48.966395 13.430375<br>European Beech (Fagus sylvatica) Belauer See 54.06061 10.15373<br>European Beech (Fagus sylvatica) Berchtesgaden 47.56574 12.89274<br>European Beech (Fagus sylvatica) Harz 51.838522 10.635239<br>European Beech (Fagus sylvatica) Pfälzerwald 49.145482 7.713357<br>European Beech (Fagus sylvatica) Scheyern 48.487405 11.428479<br>European Beech (Fagus sylvatica) Solling 51.73638 9.57365<br>Bladderwrack (Fucus vesiculosus) Eckwarderhörne 53.519772 8.231447<br>Bladderwrack (Fucus vesiculosus) Ostsee 54.506479 13.284812<br>Bladderwrack (Fucus vesiculosus) Sylt 55.011 8.4125<br>Blue Mussel (Mytilus edulis) Eckwarderhörne 53.519772 8.231447<br>Blue Mussel (Mytilus edulis) Ostsee 54.275622 12.318046<br>Blue Mussel (Mytilus edulis) Sylt 55.011 8.4125<br>Lombardy Poplar (Populus nigra) Leipzig 51.353013 12.404477<br>Lombardy Poplar (Populus nigra) Saartal 49.22591 7.00576<br>Norway Spruce (Picea abies) Bayerischer Wald 48.966921 13.435102<br>Norway Spruce (Picea abies) Belauer See 54.10381 10.24531<br>Norway Spruce (Picea abies) Berchtesgaden 47.56113 12.89025<br>Norway Spruce (Picea abies) Harz 51.792691 10.645409<br>Norway Spruce (Picea abies) Pfälzerwald 49.0902 7.43575<br>Norway Spruce (Picea abies) Scheyern 48.487405 11.428479<br>Norway Spruce (Picea abies) Solling 51.787381 9.610741<br>Zebramussel (Dreissena polymorpha) Bimmen 51.858614 6.073054<br>Zebramussel (Dreissena polymorpha) Blankenese 53.556886 9.80897<br>Zebramussel (Dreissena polymorpha) Cumlosen 53.03962009 11.63766977<br>Zebramussel (Dreissena polymorpha) Jochenstein 48.566659 13.60556<br>Zebramussel (Dreissena polymorpha) Koblenz 50.34718 7.60211<br>Zebramussel (Dreissena polymorpha) Prossen 50.92708 14.11624<br>Zebramussel (Dreissena polymorpha) Rehlingen 49.371943 6.69865<br>Zebramussel (Dreissena polymorpha) Ulm 48.33667 9.93357<br>Zebramussel (Dreissena polymorpha) Zehren 51.26445 13.4026
Access & import/export	Habitat access and permits are managed by the German Environmental Specimen Bank.
Disturbance	No disturbance was noticed.

Reporting for specific materials, systems and methods

We require information from authors about some types of materials, experimental systems and methods used in many studies. Here, indicate whether each material, system or method listed is relevant to your study. If you are not sure if a list item applies to your research, read the appropriate section before selecting a response.

Materials & experimental systems

n/a	Involved in the study
☒	☐ Antibodies
☒	☐ Eukaryotic cell lines
☒	☐ Palaeontology and archaeology
☐	☒ Animals and other organisms
☒	☐ Clinical data
☒	☐ Dual use research of concern
☐	☒ Plants

Methods

n/a	Involved in the study
☒	☐ ChIP-seq
☒	☐ Flow cytometry
☒	☐ MRI-based neuroimaging

Animals and other research organisms

Policy information about studies involving animals; ARRIVE guidelines recommended for reporting animal research, and Sex and Gender in Research

Laboratory animals	The study did not involve laboratory animals.
Wild animals	Dreissena polymorpha and Mytilus edulis were collected and stored above liquid nitrogen in the field. The GESB dissects frozen soft tissue including respiratory water and separates it from the shell. The soft tissue incl. respiratory water is ground to a fine powder. Samples are always stored below -130°C. For details see methods section in the manuscript.
Reporting on sex	No information on the sex is recorded.
Field-collected samples	Dreissena polymorpha and Mytilus edulis were collected in the field and immediately stored above liquid nitrogen.
Ethics oversight	No ethical approval or guidance was needed since the samples were collected by the German Environmental Specimen Bank which is authorized by the Central Environmental Agency of Germany.

Note that full information on the approval of the study protocol must also be provided in the manuscript.

Plants

Seed stocks	For detailed information of collection location see part "Methods - Specimen Bank Data". Plant specimen were collected according to highly standardized guidelines by the German Environmental Specimen Bank which can be found on the homepage.
Novel plant genotypes	-
Authentication	-
